# Community-Acquired Pneumonia Requiring Hospitalization among French Guianese Children

**DOI:** 10.1155/2021/4358818

**Published:** 2021-12-22

**Authors:** Alexandre Cannesson, Narcisse Elenga

**Affiliations:** Cayenne Andrée Rosemon Hospital, French Guiana, Pediatric Medicine and Surgery, Rue des Flamboyants, BP 6006, 97306 Cayenne Cedex, French Guiana, France

## Abstract

Community-acquired pneumonia remains a leading cause of hospitalizations among children worldwide. The diagnosis is based on the history, the physical examination results in children with fever plus respiratory signs and symptoms, and chest radiography. The microbiological etiology is confirmed by viral testing and hemocultures. The most likely etiology depends on the age of the child. The features of childhood pneumonia vary between countries and territories. The purpose of this study was to describe the epidemiological characteristics and current microbial ecology of community-acquired pneumonia in children in French Guiana. We performed a retrospective, descriptive, and monocentric study between January 1, 2015, and December 31, 2017, in the pediatric ward of the Cayenne Hospital in French Guiana. The studied population was aged from 0 to 15 years and 3 months and hospitalized for acute community-acquired pneumonia. A total of 415 patients (mean age 3.62 years) were included. A pathogen was identifiable in 22.4% of cases, including bacteria in 61.3%, viruses in 43%, and coinfections in 14%. The main pathogens were respiratory syncytial virus (31.2%), *Streptococcus pneumoniae* (20.4%), *Haemophilus influenzae* (11.8%), and *Mycoplasma pneumoniae* (10.8%). The burden of hospitalization for children with community-acquired pneumonia was highest among less than 2 years, in whom respiratory viruses were the most commonly detected causes of pneumonia. The share of vaccine-preventable diseases (S. pneumoniae, H. influenzae, and influenza) remains high. With the vaccination requirement imposed since 1 January 2018 against pneumococcus, Haemophilus influenzae, and whooping cough and the possibility of practicing multiplex PCR in our hospital, it will be interesting to study the impact of this law enforcement on new child generations and compare these new data to our study.

## 1. Introduction

Community-acquired pneumonia (CAP) is a leading cause of hospitalizations among children worldwide [1]. It remains a common cause of morbidity and mortality worldwide, even in industrialized countries, and its incidence is highest among children under 5 years of age [2]. The features of childhood pneumonia vary between countries and territories [1]. The knowledge of the local germs responsible for CAP is crucial to develop therapeutic strategies such as suitable probabilistic antibiotherapy but also to guide public health priorities [3]. Appropriate management of childhood pneumonia can significantly reduce pneumonia-specific mortality [4]. French Guiana has several climatic and geographic characteristics: equatorial rainforests covering 95% of the territory, a humid equatorial climate with a relatively dry and slightly warmer season from July to November, and a rainy season from December to June. With its geographical situation, its impressive biodiversity is a source of many pathogenic strains and a population that is constantly changing because of migratory flows from neighboring countries [5, 6]. These factors, combined with the many risks of exposure to contamination (displacement, nature of the habitat) [7], make it a geographical area with high potential for infectivity and at risk of emergence of new pathogens [6, 8]. Infectious diseases represent one of the first reasons for seeking care in the Offshore Centers for Prevention and Care [6], and acute febrile respiratory syndromes are among the first three causes of these consultations. At the pediatric level, infectious diseases are one of the leading causes of death among children in French Guiana [6, 9]. In this study, our main objective was to describe the epidemiology and microorganisms responsible for CAP in children hospitalized at the Cayenne General Hospital.

## 2. Materials and Methods

### 2.1. Study Design, Setting and Participants

This monocenter retrospective study was conducted on children hospitalized for CAP. This study included patients from 30 days to 15 years and 3 months of age. Medical charts from all patients were identified using the Pediatric Hospital Information System with the International Classification of Diseases, 10th edition, with a discharge code of pneumonia, from January 1, 2015, to December 31, 2017. Patients hospitalized with a nosocomial infection were excluded (*n* = 11). In addition, patients were excluded if no medical record information was available (*n* = 11) and if, after a thorough review of the file, the diagnosis of pneumonia was incorrect (*n* = 8). Patients meeting the inclusion criteria (*n* = 415) were categorized into 3 mutually exclusive categories: (i) those with documented microorganisms on blood culture or pleural fluid culture; (ii) those without microorganisms documented by a negative or contaminated blood culture or a negative virological research by polymerase chain reaction (PCR), on nasopharyngeal aspiration; and (iii) those in whom a blood culture, any other culture, or PCR had not been performed (no culture). All culture data were obtained within 48 hours of hospital admission.

### 2.2. Data Source

Detailed medical record reviews were performed in all children in this study. Collected data included demographics, presenting signs and symptoms, physical examination findings, and laboratory, radiograph, and microbiology results, including bacterial pathogens and antibiotic susceptibility patterns, viral and parasitic pathogens, and PCR. The following clinical outcomes were also recorded: length of stay, presence of pneumonia-associated sequelae, supplemental oxygen requirement, and intensive care or resuscitation unit's admission. Treatment data during hospitalization and discharge antibiotic therapy were also collected. Patients were identified by a unique number. Data from the medical record review were entered into an Excel anonymized database for analysis.

### 2.3. Ethical and Regulatory Aspects

An informed written consent to participate in the study has been obtained from each parent or legal guardian. According to the European regulation, French observational studies from data obtained routinely from patient health care records do not need the approval of the ethics committee [10]. These anonymized data issued from medical records were analyzed, which was authorized according to the regulatory authorities (Commission Nationale Informatique et Libertés (CNIL). As this anonymized hospital database has already been declared and approved by the CNIL, the ethics committee of Cayenne Hospital deemed it unnecessary to examine this request. All methods were carried out in accordance with relevant guidelines and regulations.

### 2.4. Study Definitions

CAP was categorized by the following:

- Probable bacterial pneumonia: (i) consolidated pneumonia according to the World Health Organization (WHO) criteria for interpretation of chest X-rays [11] and (ii) pneumonia with pleural effusion or bacterial confirmed pneumonia (isolation of *S. pneumonia* or *Haemophilus influenzae* in blood or pleural fluid, regardless of the type of infiltrate)
- Nonbacterial pneumonia: chest X-rays with interstitial infiltrate or without any pathological finding

Organ dysfunction was based on consensus pediatric guidelines [12]. Outcome was categorized as a good outcome or a poor outcome (poor outcome = transfer to the resuscitation unit and/or bad evolution to death).

### 2.5. Laboratory Diagnosis

For bacterial etiological diagnosis, blood cultures and pleural fluid cultures (if the patient required thoracocentesis) were performed in all hospitalized patients.

The parasite and fungal infections were also diagnosed by blood or pleural fluid cultures.

A nasopharyngeal aspiration was performed in case of epidemiological and/or clinical suspicion of a respiratory viral infection.

Pathogenic bacteria included *Streptococcus pneumoniae*, *Staphylococcus aureus*, and *Haemophilus influenzae*. Bacteria considered as contaminants (coagulase-negative *Staphylococcus* spp, *α*hemolytic *Streptococcus* spp, *Corynebacterium* spp, *Bacillus* spp, and *Micrococcus* spp.) were considered as pathogenic as they were found in blood cultures.

Antimicrobial therapy was recorded daily and initially consisted of penicillin/ampicillin with or without an aminoglycoside and co-amoxyclavulanic acid and third-generation cephalosporin as second-line therapies (13, 14).

Blood culture–directed changes in antibiotics were classified as broadened, narrowed, or unchanged relative to *Streptococcus pneumoniae* and *Staphylococcus aureus*.

### 2.6. Statistical Analysis

The characteristics of the study population were described overall and within their outcome groups. Continuous variables were summarized using median and interquartile range (IQR) and compared between children with good outcomes and poor outcomes using the Wilcoxon rank-sum test. Categorical variables were described using counts and frequencies and compared between those with good outcome and poor outcome using the *χ*^2^ test or Fisher's exact test. The prevalence of bacteremia and other etiologies was determined for all patients, patients with good outcome and poor outcome, using binomial exact 95% confidence intervals. Statistical analysis was performed using Stata 15.

## 3. Results

**Figure 1:**
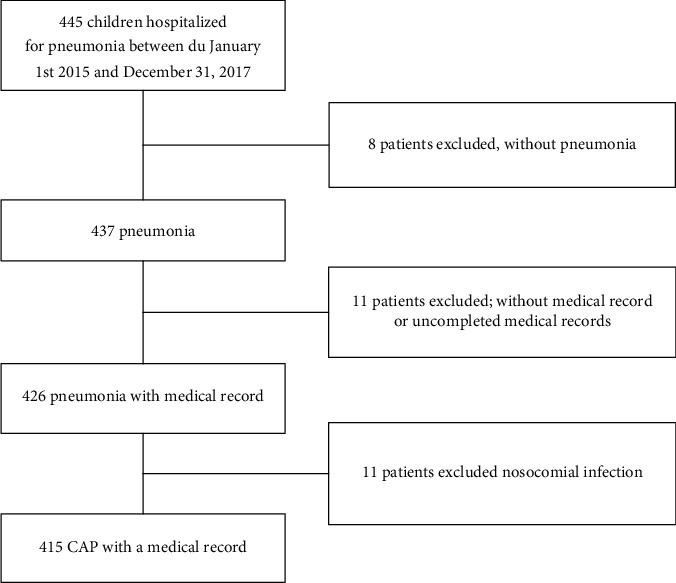
Flow chart of the patients included in the study.

Overall, 415 patients were finally included in this study (Figure 1). Table 1 presents the general characteristics of our study population. The mean age was 3.5 ± 3.0 years. Fifty-two percent and 74.7% of the patients were less than 2 years and 5 years old, respectively. The mean birth weight was 2.85 ± 0.80 kg. 27.8% of the patients were premature (among them, there were 18.6% of average prematurity, 3.9% of great prematurity, and 5.4% of considerable prematurity). 55.9% were male. The origins of our population were mainly consultations (74.5%), 6.7% transferred from Saint-Laurent-du-Maroni or Kourou, and 18.8% from the inside area of French Guiana. The immunization was up to date for only 52.8% of our study population, with 46.3% of non-up-to-date vaccinations against *Streptococcus pneumoniae*. In regard to risk factors, 14.4% of our patients were asthmatic or had a history of three episodes of bronchiolitis before the age of 3 years.

Regarding other comorbidities,

- 10.6% of the patients were sickle cells (SS, SC, or S-beta thalassemia)
- 4.1% had congenital heart disease
- 3.8% had bronchopulmonary dysplasia (hyaline membrane disease)
- 2% had trisomy 21 or 18, Alagille disease, or Pompe disease

These comorbidities (34.2%) concerned only 123 patients because 14 of them had been hospitalized several times during the 3 years of study. 25.2% of our patients had received antibiotics before hospitalization, for most of them penicillin. 24.1% of them have been hospitalized in the previous 6 months (hospitalization at birth included).

The average consultation time was 2.3 ± 2 days. 53.4% of the patients had fever at home and 48% at the initial clinical examination (emergency or consultation) with an average temperature of 102.74°F.

**Figure 2:**
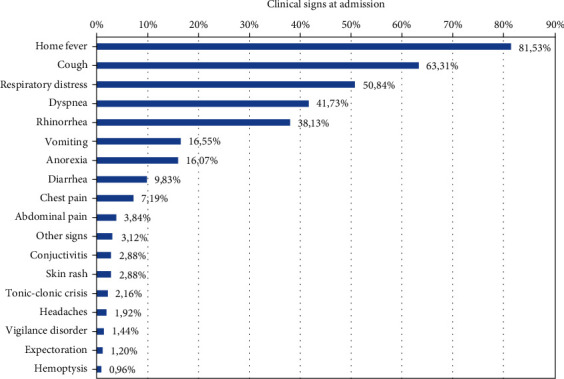
Clinical signs at admission.

The average heart rate was 146 ± 29.7 per minute, and the average respiratory rate 44 ± 14.6 per minute. The median oxygen saturation in ambient air was 95.2%. Auscultation was abnormal in 74.3% of cases: crackling rattles (43.07%). 98.6% of patients had an initial chest X-ray. Among radiographs performed (387 patients), condensation (60.9%), infiltration (20.4%), or effusion (4.4%) was mostly visible. Radiography was interpreted as normal (clinical diagnosis of pneumonia) in 69 patients (17.8%). The bilateral image rate was 27.8% of cases (108 patients), with 4 effusions among them (Figure 2).

**Figure 3:**
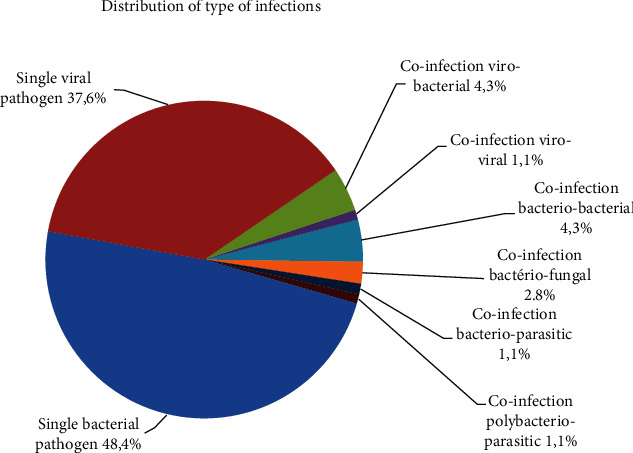
Distribution of type of infections.

The etiology of CAP was identified in 93 cases (22.4%). Among them, 41.2% had no specific identification exam (except bacterial blood cultures and urinalysis) and 88 patients without identified microorganisms have been treated with antibiotics before hospitalization (against 15 patients with an etiology). A bacterial etiology was found in 57 cases, out of a total of 93 blood cultures (61.3%) (45 patients with a single bacterium, four with bacterial and viral coinfection, four with bacteriobacterial coinfection, two with bacteriofungal coinfection, one with bacteriospore infection, and, finally, one patient with a bacterioparasitic coinfection) (Figure 3). A viral etiology was found in 40 cases (43%) (35 patients with a single viral term, 4 patients with bacterioviral coinfection, and 1 patient with viral coinfection). Coinfections accounted for 13 patients, representing 14% of patients with identified pathogens (Figure 3). Among the 17 pleural effusions on radiography, 8 bacterial origins were found (47%): one *Staphylococcus epidermidis*, two *Mycoplasma pneumoniae*, one *Staphylococcus aureus*, three *Streptococcus pneumoniae*, and one *Klebsiella pneumoniae*; and there was no germ found for the 9 others.

### 3.1. Pathogens Identified according to the Year of Hospitalization

**Figure 4:**
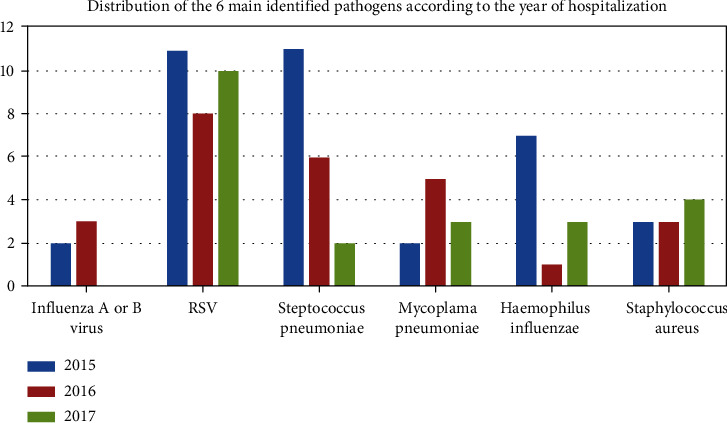
Distribution of the 6 main identified pathogens according to the year of hospitalization.

There were 106 pathogens identified in 93 patients. RSV and *Staphylococcus aureus* levels remained stable during the 3 years of study (Figure 4). However, there was a decrease in the number of *Streptococcus pneumoniae* during these 3 years (11 in 2015, six in 2016, and two in 2017), on a hundred samples per year.

### 3.2. Identification of Pathogens according to Age (Figure 5)

In our study, the vast majority of RSV was found in patients less than 2 years old (28 patients versus one patient between 2 and 5 years).

### 3.3. Pathogens Identified according to Seasonality and Meteorology (Figures 6)

The RSV was more present during the months of January and March. *Streptococcus pneumoniae* was more present during the months of January, February, and September. The number of hospitalized patients appears to be correlated with rainfall during the months of May, June, and July.

### 3.4. Pathogens Identified according to Vaccination Coverage

Of the 19 *Streptococcus pneumoniae*, information on pneumococcal vaccination was available for 14 patients. Among them, 11 were not up to date: 78.6% (no vaccination or recall (s) not made). Among the 11 patients with *Haemophilus influenzae*, immunization information was available for 9.

### 3.5. Biology and Prognostic Factors according to the Identified Pathogen

In patients with an identified bacterium, the average white blood cell count was 17.31 ± 9.23 G/L with 10.73 ± 8.30 G/L neutrophils. The average CRP was 101.1 ± 10.8 mg/L and PCT 2.76 ± 1.04 *μ*g/L. In patients with an identified virus, the mean leukocyte count was 12.00 ± 5.44 G/L with 6.18 ± 4.34 G/L neutrophils. Their average CRP was 24.20 ± 4.19 mg/L and PCT 0.68 ± 0.43 *μ*g/L. These calculations did not take into account bacterioviral coinfections.

### 3.6. The Prognostic Factors according to the Identified Pathogen

**Figure 7:**
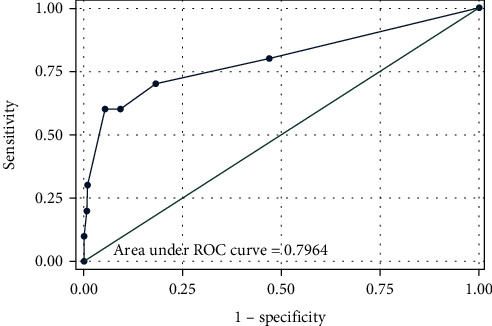
ROC curve testing our multivariate model. The area under the ROC curve was close to 1, confirming the quality of the model. The ROC analysis is used here to quantify how accurately our medical diagnostic test (bacterial pneumonia CRP > 130 mg/L) can discriminate between poor and good outcomes in children hospitalized for CAP.

The multivariate analysis showed that patients who have bacterial pneumonia and CRP > 130 mg/L were at risk of bad outcome. 98% of patients without these anomalies had a favorable evolution (Figure 7).

### 3.7. Antibiogram and Resistance Profile of the Identified Bacteria

Of the nine strains of *Haemophilus influenzae*, four showed decreased susceptibility to amoxicillin and 4 to cefalotin. And three of the seven strains tested exhibited decreased susceptibility to rifampicin. Of the six strains of *Streptococcus pneumoniae*, two had decreased susceptibility to amoxicillin, two to gentamicin, two to erythromycin, and two to tetracycline. And two of the five strains tested showed decreased sensitivity to trimebutine sulfadiazine. Two of the eight strains tested for *Staphylococcus aureus* exhibited decreased susceptibility to oxacillin, cefotaxime, and erythromycin. And three of these eight strains exhibited diminished sensitivity to tetracycline.

### 3.8. Therapeutic Management and Outcome of Hospitalization

172 patients were treated with amoxicillin 46.2%, 92 (24.7%) with amoxicillin+clavulanic acid, 113 (30.4%) with cefotaxime, 40 (10.8%) with ceftriaxone, 100 (26.9%) with azithromycin, 21 (5.6%) with josamycin, 10 (2.7%) with clarithromycin, 12 (3.2%) with ciprofloxacin, 88 (23.7%) with gentamycin, 39 (10.5%) with vancomycin, 20 (5.4%) with rifampicin, and 14 (3.8%) with clindamycin in case of severe staphylococcal pneumonia. The mean duration before apyrexia was 2.14 ± 1.6 days with a mean duration of antibiotic therapy of 10.4 ± 2.6 days. The mean total length of stay was 7.35 ± 2. 17 (4.1%) patients required a transition to the resuscitation unit among the 110 patients in the intensive care unit.

## 4. Discussion

This study allows attracting attention to some characteristics of childhood CAP in French Guiana. 45.3% of our patients had comorbidities, mostly history of preterm birth (25.5%), asthma (14.4%), and sickle cell disease (10.6%). Vaccination was up to date for only 52.8% of our study population. An etiology was found in 93 patients (22.4%). A bacterial origin was found in 57 (61.3%) of these patients with 40 (43%) with a viral origin. This low detection of bacteria could be explained by the fact that some children (*n* = 88, 21%) had been treated with antibiotics before hospitalization. However, as part of routine care, in the event of a bronchiolitis or influenza epidemic, virological testing is not performed in all children, which might be the reason why the virus detection rate is lower than that of bacteria. There were 13 coinfections (14%) with identified pathogens. The rate of pathogens identified in studies comparable to ours is 20 to 60%, according to the diagnostic possibilities [13, 14]. In adults, pathogens remain unknown in more than 50% of hospitalized CAP, despite bacteriological cultures, serology, research by PCR, or rapid immunochromatography tests [15]. However, new PCR techniques, called multiplexes, have improved the yield diagnostic so that a pathogen can be detected in 65 to 86% of the patients [16–19]. However, a study similar to ours, conducted in children aged 2 to 59 months (406 children) with severe pneumonia in Quito, Ecuador, in 2017 [20], found 39.2% RSV. Concerning bacterial agents, our study is in agreement with the literature of European countries and the United States [21].

Our study highlights the impact of the low vaccination coverage rate in French Guiana. However, this argument does not seem to be sufficient to explain the difference with the other countries of Latin America and the Caribbean, since these countries also do not have an optimal vaccination coverage rate. According to Jiang et al. [19], coinfections represented 34.6% of patients with a majority of 71.3% virobacterial coinfections. Jonnalagadda et al. [20] found 25.6% of coinfection, viroviral in majority (19.4% of RSV and metapneumovirus). Other recent studies in children have found viral and bacterial codetection in more than 25% of CAP cases [22, 23]. Recent meta-analyses of the etiological data suggested that clinical pneumonias are caused by the sequential or simultaneous interaction of more than one microorganism. Indeed, the composition of the nasopharyngeal microbiota is constantly subject to interactions between species. It is now clearly established that microbial interactions are multifactorial and involve a complex interaction between multiple host factors and bacterial characteristics. This may have important consequences for the composition and stability of the microbial community itself and susceptibility to disease [24]. It is also plausible that viral presence is thought to predispose the respiratory niche to bacterial colonization by different known mechanisms [24]. In particular, severe CAP is often caused by multiple pathogens [25, 26]. Moreover, in our study, the average length of stay in patients with comorbid identified infection was 13.3 days, almost double of that of our overall population (7.6 days). We have noted some Guianese specificities such as *Plasmodium vivax*, *Cryptococcus neoformans*, or hookworm parasite in coinfection with a bacterium. There was no identification of Q fever or Amazonian toxoplasmosis, two pathogens that are the main causes of pneumonia in adult patients in French Guiana. Indeed, in French Guiana, Q fever pneumonia represented 24.4% of CAP adult inpatients [27]. Regarding the etiology according to the age of the patient, our data are entirely in agreement with the literature for children under 5 years old [23–25]. However, the results obtained from our study should be interpreted with caution because of methodological limits. The retrospective nature of the study inevitably constituted a limiting factor for the data collection, particularly concerning the history and the initial clinical condition. Our records did not allow us to study other known risk factors such as indoor air pollution or household air pollution from solid cooking fuels that might play a role in French Guiana. “Real-life” care, following recommendations and without etiological systematic research by all available microbiological methods, decreased consequently the number of germs identified and therefore their subsequent analyses [28–30]. The presented approach, however, provided additional information on the actual support and current hospitalized children in Cayenne Hospital. The monocentric nature of this study also allowed for a bias. The fact that Cayenne Hospital is the referral center in French Guiana probably contributed to increase artificially the number of the most serious patients. The data related to the care of these patients were probably overestimated (morbidity and mortality, average length of stay, antibiotic duration, etc.). Despite its limitations, this first study on childhood CAP in French Guiana exhaustively compares our data with those of published works. Most of our results are consistent with those of Latin America and the Caribbean, confirming the predominance of viral causes before two years of age and a significant proportion of vaccine-preventable diseases. Unlike the adult patient, we did not find any CAP caused by Q fever or Amazonian toxoplasmosis. This study will serve as a frame for a more comprehensive exploration and analysis protocol in the future.

## 5. Conclusion

To date, this article provides the most comprehensive analysis of the contributing causes of CAP in children in French Guiana. With the vaccination requirement imposed since 1 January 2018 against *Pneumococcus*, *Haemophilus influenzae*, and whooping cough and the possibility of practicing multiplex PCR in our hospital, it will be interesting to study the impact of this law enforcement on new child generations and compare these new data to our study.

## Figures and Tables

**Figure 5:**
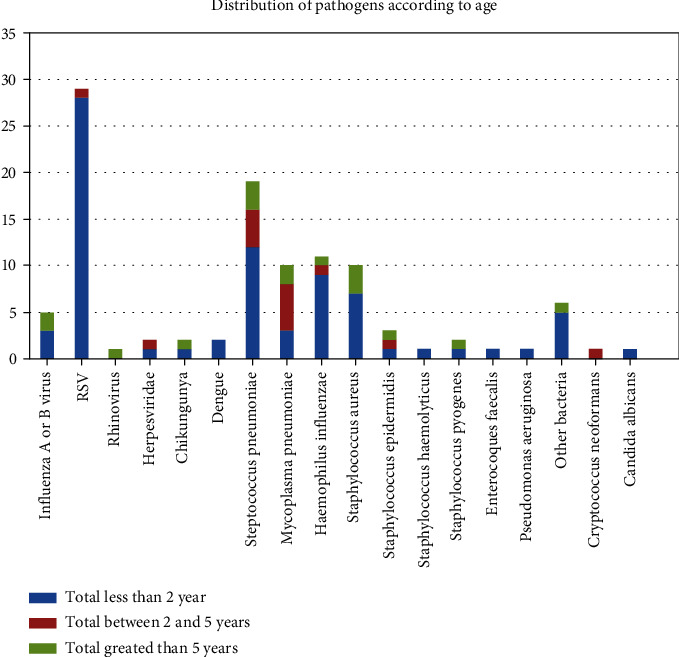
Distribution of pathogens according to age.

**Figure 6:**
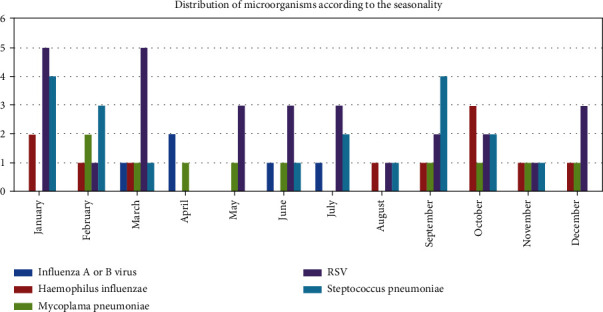
Distribution of microorganisms according to the seasonality.

**Table 1:** Univariate analysis of predictive factors of poor outcome.

Variable	Poor outcome	Good outcome	Odds ratio (IC 95%)	*p*
Age (median, IRQ)	1.4 [0.2-13]	1.8 [0.8-5]	0.6 [0.3-1.2]	0.1
Sex (male)	6 (60)	245 (56)	1.1 [0.3-4.2]	0.8
Prematurity	3 (30)	82 (19)	1.8 [0.5-7.3]	0.4
Sickle cell disease	1 (10)	44 (10)	0.9 [0.1-7.9]	0.9
Other comorbidities	0 (0)	79 (18)		
Bacterial infection	6 (60)	61 (14)	9.1 [2.5-33.4]	0.001
Viral infection	0 (0)	42 (10)		
Anemia (Hb < 10 g/dL)	11 [10-12]	11 [9.8-12]	0.7 [0.2-3.4]	0.6
CRP (median, IRQ)	132.2 [5-178.2]	44.9 [15.3-103.8]	3 [0.9-11]	0.09
PCT (median, IRQ)	0.26 [0.18-0.32]	1.34 [0.23-8.23]	0.1 [0.03-0.50]	0.003
Lactates (median, IRQ)	3.3 [2.6-4.4]	2.5 [1.7-4.0]	0.7 [0.1-5.8]	0.7

## Data Availability

Our database is available from the corresponding author on reasonable request.

## Abbreviations

RCPC: Relocated Centers for Prevention and Care
HIV: Human immunodeficiency virus
AIDS: Acquired immune deficiency syndrome
CAP: Community-acquired pneumonia
PCR: Polymerase chain reaction
IQR: Interquartile range
CNIL: Commission Nationale Informatique et Libertés
CRP: C-reactive protein.
